# Alzheimer’s Disease Animal Models: Elucidation of Biomarkers and Therapeutic Approaches for Cognitive Impairment

**DOI:** 10.3390/ijms22115549

**Published:** 2021-05-24

**Authors:** Tsuyoshi Nakai, Kiyofumi Yamada, Hiroyuki Mizoguchi

**Affiliations:** 1 Department of Neuropsychopharmacology and Hospital Pharmacy, Nagoya University Graduate School of Medicine, Nagoya 466-8560, Japan; t-nakai@med.nagoya-u.ac.jp (T.N.); kyamada@med.nagoya-u.ac.jp (K.Y.); 2 Medical Interactive Research and Academia Industry Collaboration Center, Research Institute of Environmental Medicine, Nagoya University, Nagoya 464-8601, Japan

**Keywords:** Alzheimer’s disease, amyloid cascade hypothesis, tau, neurofibrillary tangles, biomarkers, animal models, pharmacological intervention

## Abstract

Alzheimer’s disease (AD) is an age-related and progressive neurodegenerative disorder. It is widely accepted that AD is mainly caused by the accumulation of extracellular amyloid β (Aβ) and intracellular neurofibrillary tau tangles. Aβ begins to accumulate years before the onset of cognitive impairment, suggesting that the benefit of currently available interventions would be greater if they were initiated in the early phases of AD. To understand the mechanisms of AD pathogenesis, various transgenic mouse models with an accelerated accumulation of Aβ and tau tangles have been developed. However, none of these models exhibit all pathologies present in human AD. To overcome these undesirable phenotypes, *APP* knock-in mice, which were presented with touchscreen-based tasks, were developed to better evaluate the efficacy of candidate therapeutics in mouse models of early-stage AD. This review assesses several AD mouse models from the aspect of biomarkers and cognitive impairment and discusses their potential as tools to provide novel AD therapeutic approaches.

## 1. Introduction

Dementia is defined as a syndrome, caused by various brain disorders, that affects memory, thinking, behavior, and the ability to perform daily activities [1]. Worldwide, there are approximately 50 million people living with dementia. Alzheimer’s disease (AD) accounts for 60–80% of these cases [2]. AD is an irreversible and progressive neurodegenerative disorder that presents with cognitive impairment and memory loss as the primary clinical symptoms. The disease is mainly caused by the accumulation of extracellular amyloid β (Aβ) and intracellular neurofibrillary tau tangles [3]. Structural brain changes are thought to begin 20 years or more before the onset of clinical symptoms [4]. The disease has several phases, which include a long preclinical phase with no clinical symptoms, a mild cognitive impairment phase, and a disease phase [5]. The length of each phase is affected by the patient’s age, genetics, gender, and other factors [6]. Although AD has been studied for over 100 years, fundamental treatment strategies for the disease remain underdeveloped. It is therefore important to develop new therapeutic medications for AD patients that are more effective than the existing treatments.

Rodent models are evaluated using behavioral tests such as the Morris water maze (MWM), the radial maze, the Y-maze, the T-maze, fear conditioning (FC), and novel object recognition (NOR) tests. These tests play a crucial role as indicators of learning, memory, and cognitive functions, which correspond to the late phases of cognitive deficits in AD patients [7,8]. This review summarizes the characteristics of several representative AD mouse models that were generated based upon the amyloid cascade hypothesis. In addition, we assess these models in regard to changes in biomarkers, pathologies, and behavior, and we discuss their potential as useful tools to provide therapeutic approaches in AD. In conjunction with this analysis, we introduce several approaches to develop new therapeutic strategies for AD.

## 2. Amyloid Cascade Hypothesis

The amyloid cascade hypothesis was first proposed in 1991 by Hardy and Allsop [9]. They identified a pathogenic mutation in the *Aβ precursor protein* (*APP*) gene on chromosome 21 in familial AD (FAD) patients. The mutation causes APP mismetabolism and Aβ deposition, which are primary events in AD disease progression. They further suggested that the pathology of AD is caused by abnormal fibrous protein deposits, including senile plaques and neurofibrillary tangles (NFTs), and amyloid deposits on the walls of cerebral blood vessels. Senile plaques and cerebrovascular amyloids are caused by the deposition of extracellular Aβ. Aβ first isolated from the meningeal vessels of AD patients by Glenner and Wong in 1984 [10], is a 39–43 residue protein derived from multiple proteolytic cleavages of APP. APP was first cloned and sequenced in 1987 [11,12], is 695 amino acid residues in length, and may function as a glycosylated receptor on the cell surface [13]. APP is degraded by the proteolytic enzymes α-, β-, and γ-secretase in two processing pathways [14]: Aβ–non-producing and Aβ-producing. In the Aβ–non-producing pathway, APP is hydrolyzed by α-secretase and then by γ-secretase, which has presenilin 1 (PSEN1) as the core active subunit. ADAM9, ADAM10, and ADAM17 may also have α-secretase activity [15]. Importantly, these cleavage events do not lead to Aβ deposition in the brain because this pathway does not produce insoluble Aβ. By contrast, in the Aβ-producing pathway, APP is hydrolyzed by β-secretase (BACE1) followed by γ-secretase, which leads to the production of insoluble Aβ [14]. The levels of insoluble Aβ may play an important role in the onset and progression of AD [16]. As APP hydrolysis mainly occurs through the Aβ–non-producing pathway, insoluble Aβ protein is not produced under normal conditions. The small amount of APP hydrolyzed via the Aβ-producing pathway is usually eliminated by the immune system. Aβ may function as a neurotrophic factor for differentiating neurons, but the high concentrations of Aβ in AD patients cause neuronal death [17]. Moreover, cholinergic and noradrenergic neurons are highly sensitive to amyloid toxicity, resulting in a decrease in these neurons in the preclinical phase, well before the appearance of amyloid plaques and cognitive deficits [18,19]. In FAD patients harboring APP mutations near the BACE1 cleavage site, APP is likely hydrolyzed by the Aβ-producing pathway [20,21,22], resulting in excessive accumulation of insoluble Aβ and the eventual development of AD. Additionally, two Aβ peptides are formed by BACE1 and γ-secretase cleavage events: a peptide terminating at amino acid residue 40 (Aβ40) and a longer form ending at residue 42 (Aβ42) [16,23]. Aβ42 is more likely to misfold and aggregate than Aβ40, suggesting that Aβ42 is more neurotoxic [24]. Aβ42 plasma levels are elevated in AD patients, suggesting that this increase is associated with the development of AD [25]. 

## 3. Tau Propagation Hypothesis

The formation of intracellular NFTs, derived from microtubule-associated tau, is also a hallmark of AD pathology. The tau propagation hypothesis was introduced in 2009 [26]. Tau, a 352 residue thermostable protein, was identified in 1975 [27] and cloned and sequenced in 1988 [28]. Tau stabilizes microtubules, thereby promoting the polymerization of tubulin into microtubules [29]. Although phosphorylated tau maintains the cytoskeleton, neuronal tau may regulate the stability of axonal microtubules and eventually transports signal transduction–associated proteins through the microtubules. In the human brain, six predominant tau isoforms are formed by selective splicing [30]. These isoforms are distinguished by the presence or absence of two N-terminal inserts (exon 2 and 3 inclusion) and the presence of either three or four imperfect arginine repeats (3R or 4R) in the microtubule binding domain at the C-terminus (exon 10 inclusion) [31]. These observations suggest that a balanced tau isoform ratio is necessary for maintaining normal brain function in humans, and an imbalance in these ratios would therefore lead to tau aggregation into NFTs. In humans, tau pathology is observed initially in a specific area of the brain and then spreads to other areas [32]. Although tau has 85 potential serine, threonine, and tyrosine phosphorylation sites [33], abnormal phosphorylation at approximately 45 different sites is related to AD pathology [34]. Excessive or abnormal phosphorylation may be caused by alterations in the activities of various kinases or phosphatases that target tau. Abnormally phosphorylated tau forms anti-helical fibrils and depolymerizes microtubes, which is followed by the formation of insoluble NFTs [35]. The insoluble NFTs may impair cytoplasmic function and interfere with axonal transport between neurons, thereby inducing cell death. The molecular and cellular mechanisms responsible for the formation of tau lesions remain unclear.

## 4. Biomarkers of AD

Based on the amyloid cascade and tau propagation hypotheses, the measurement of Aβ40, Aβ42, and tau in cerebrospinal fluid (CSF) was established as diagnostic for AD biomarkers. A decrease in CSF Aβ42 levels [36], occurring before AD onset [37], and elevated tau levels [38,39,40] were observed in AD patients. However, an increase in CSF tau levels was also detected in other neurological diseases, including psychiatric disorders [38,39,40] and normal aging [38,39]. For this reason, it was deemed advantageous to combine the measurement of Aβ40, Aβ42, and tau in CSF to confirm AD in patients, and it was hoped that these measurements would be a useful biological marker for AD therapeutic strategies [41]. In addition, Aβ oligomers in CSF may play a critical role in the pathogenesis and progression of AD [42,43]. However, the measurement of CSF biomarkers is a highly invasive test, and the physical burden on the elderly is particularly high. Positron emission technology (PET) using ^11^C-labeled Pittsburgh Compound B (^11^C-PIB) to visualize amyloid accumulation in the brain of AD patients was developed [44,45]. Aβ PET radiotracers enable detection of early-stage AD pathology [46]. Consequently, amyloid PET is widely used to diagnose preclinical AD. ^18^F-Florbetapir, approved by the United States Food and Drug Administration (FDA), was the first ^18^F-labeled tracer developed to detect Aβ in ^18^F-fluorodeoxyglucose (^18^F-FDG) PET [47]. Although ^18^F-Florbetapir is the most widely used Aβ tracer, ^11^C-PIB has a higher binding affinity for amyloid and a shorter half-life. As an amyloid PET scan can only be performed in facilities that are equipped with a cyclotron and a device for preparing labeled compounds, this diagnostic is often cost-prohibitive. Recently, PET imaging that detects tau accumulation [48,49] or microglia activation [50,51] was also developed. Amyloid PET scans are expensive and CSF collection is invasive, therefore the development of tests for measuring AD biomarkers from blood samples was highly anticipated. Although it is difficult to detect biomarkers in human blood [52,53,54], mass spectrometry [55,56,57] and enzyme-linked immunosorbent assay [58] successfully detect amyloid in peripheral blood. Determination of the alteration of Aβ secondary structure in blood plasma by an immune infrared sensor is also possible, suggesting that this change could be used as a blood biomarker for severe AD stages [59]. Additionally, the usefulness of measuring tau [60,61,62], Interleukin-8 [63], and neurofilament light [64] levels in blood plasma are confirmed in preclinical AD. The analysis of microRNAs in blood was also shown to be effective for prospective AD risk prediction, suggesting that microRNAs may be of practical clinical use as biomarkers of AD [65]. Moreover, signal transduction proteins, such as insulin-like growth factor (IGF) 1 and IGF-binding protein 3, may predict cognitive decline [66]. Aβ clearance proteins, including apolipoprotein (APO) A-I, complement protein C3, and transthyretin in the serum, may also be potential biomarkers for mild cognitive impairment evaluation [67]. However, it is difficult to unify the analysis techniques for these biomarkers worldwide, and these biomarker tests are not yet widespread. If AD biomarkers from blood samples can be established and used for pre-screening before expensive PET imaging, it would greatly decrease the cost of developing therapeutic or preventive drugs, consequently accelerating their development. An overview of these biomarkers can be found in Table 1.

## 5. Biomarkers and Amyloid Cascade Hypothesis-Related Animal Models

In FAD patients, accumulating evidence indicates that Aβ begins to deposit extracellularly 20–30 years before the onset of cognitive and memory impairments and before secondary tau appears in neurons. For this reason, it is widely believed that the amyloid cascade hypothesis is the main working hypothesis in AD. To investigate AD pathology, molecular mechanisms, and cognitive function, *APP* and *PSEN* gene mutations were identified in FAD patients [20,68]. Transgenic (Tg) mice that overexpresses the *APP* gene mutation associated with FAD (APP-Tg mice) were generated as AD mouse models [2]. Over the past 20 years, many additional Tg mice, carrying multiple mutations including the *APP* and *PSEN* familial gene mutations, were developed. Molecular biological studies in these models indicate that several genes and their mutations play a role in the development of early-onset AD. In addition to the *APP*, *PSEN1*, and *PSEN2* genes, more than 20 genetic risk loci for AD were identified [69]. Among these genes, a mutation in *apolipoprotein E* (*APOE*) and a rare variant of the *triggering receptor expressed on myeloid cells 2* (*TREM2*) are the most common genetic risk factors for late-onset AD. Below, we will briefly summarize the advantages and limitations of several popular Tg mice as AD mouse models. An overview of the timeline for amyloid, phosphorylated tau, and NFT pathologies observed in these APP-Tg mice can be found in Table 2.

### 5.1. Animal Models for Early-Onset AD

#### 5.1.1. Tg2576

The APPSWE (Tg2576) mice overexpress the 695–amino acid isoform of human APP protein [containing the Swedish mutation (K670N and M671L, and KM670/671NL)] under the control of the hamster prion protein (PrP) promoter [70]. Tg2576 mice show a 5-fold increase in Aβ40 and a 14-fold increase in Aβ42/43 concentrations in the old mice compared to the young mice. Inflammatory markers, such as elevated levels of cytokines and microglia activation, are observed in aged mice [71]. In behavioral tests, these mice also exhibited learning and memory impairments in spatial reference and alternation tasks at 9–10 months of age, although no significant differences were found at 3 months of age [70]. Additionally, Tg2576 mice exhibit learning- and memory-associated abnormalities in the T-maze alternation task [72] and hippocampus-dependent fear memory [72,73] and amygdala-dependent cued fear learning ability in the FC test [74], suggesting that these mice have a widespread deficit in cognitive functions. The behavioral and pathological features of Tg2576 mice resemble those observed in human AD. Alterations in adult brain neurogenesis were reported for Tg2576 mice and human AD patients [75]. In contrast to the aforementioned similarities between Tg2576 mice and human AD patients, there are several key differences. Although widespread neuronal cell loss and NFT pathology are observed in the cortex and hippocampus in advanced-stage AD patients [76], this is not recapitulated in the Tg2576 mice [77]. Brain dysfunction occurs prior to amyloid deposition in this mouse model, suggesting that the symptoms observed could be due to soluble Aβ species. 

#### 5.1.2. APP23

The APP23 mice overexpress the human APP protein with the Swedish double mutations, which combine KM670/671NL with the V717I mutation, under the control of the mouse Thy1 promoter [78]. As this mouse model has the same Swedish mutations found in the Tg2576 mouse, the neuropathological and behavioral phenotypes of APP23 mice resemble those of Tg2576. Amyloid plaques in this model are observed at 6 months of age [78], followed by neuritic and synaptic degenerations and abnormal tau phosphorylation induced by Aβ plaque deposition with aging. Neuronal loss is found in the CA1 region of the hippocampus [79] and neocortex [80] in the brains of these mice. Similar to AD patients and Tg2576 mice, APP23 mice exhibit amyloid angiopathy in the cerebral vessels [81]. Additionally, APP23 Tg mice display age-related abnormal phenotypes associated with cognitive function in various behavioral tests [82,83,84,85,86]. These phenotypes in APP23 mice are similar to all symptoms observed in AD patients. 

#### 5.1.3. PDAPP

The PDAPP mice overexpress the human APP protein with the Indiana mutation (V717F) under the control of a platelet-derived growth factor-β promoter [87,88]. Similar to AD patients, PDAPP mice show an accumulation of amyloid plaques beginning at 6–9 months of age, resulting in glial activation, dystrophic neurites, gliosis, and loss of synaptic and dendritic density in the hippocampus. PDAPP mice express high levels of human APP, more than 10-fold higher than endogenous murine APP. Unlike AD patients, PDAPP mice do not have NFTs or neuronal loss, although immunoreactivity of phosphorylated tau is observed in dystrophic neurites [89]. The PDAPP and Tg2576 mice differ in neuropathology. Tg2576 mice show a progressive increase in both Aβ40 and Aβ42 levels [70], whereas PDAPP mice do not have elevated levels of Aβ42. A number of amyloid deposits in dense-cored plaques were observed in Tg2576 mice, whereas PDAPP mice have only a few diffuse deposits [87,88]. Moreover, unlike Tg2576 mice, giant plaques and vascular amyloid depositions are also largely absent in PDAPP mice. This mouse model has cognitive and memory deficits at a young age in a variety of behavioral tests prior to Aβ plaque deposition [90,91,92].

#### 5.1.4. TgCRND8

The TgCRND8 mice overexpress a double mutant form of the human APP protein (KM670/671NL and V717F) under the control of the PrP promoter [93]. This mouse model has thioflavin S-positive Aβ deposits at 3 months of age that closely resemble those seen in AD. Dense-cored plaques and selective neuronal loss are detected at 5 months of age [93], followed by the accumulation of hyperphosphorylated tau at 7–12 months of age in the neocortex and in the dentate gyrus (DG), CA1, and CA3 regions of the hippocampus [94]. No deposition of NFTs is observed in this model [93]. Neuromorphological abnormalities do not appear in this mouse model until 6 months of age [95]. Age-related behavioral impairments in TgCRND8 mice are observed starting at 3 months of age in spatial working and reference memory tasks including the Barnes maze, MWM, NOR, and FC tests [93,96,97,98,99,100]. Therefore, this mouse model, as well as Tg2576, could be potentially powerful models for developing therapeutic drugs to treat early-onset AD. However, Aβ deposits and dystrophic neurites in TgCRND8 and Tg2576 mice are similar to those noted in human pathological aging but not to those in AD [101]. 

#### 5.1.5. APPPS1

The APPPS1 mice overexpress human APP (with the KM670/671NL mutation) and PSEN1 (with the L166P mutation), both under the control of the mouse Thy1 promoter [102]. This model has an impairment in amyloid protein processing, resulting in elevated Aβ42 levels at 2–3 months of age [102]. Cerebral amyloidosis is observed starting at 6–8 weeks-old. Formation of NFTs is not observed in this model, although hyperphosphorylated tau-positive neurites are detected at 8 months of age. Various pathological abnormalities, including glial activation [103], dystrophic neurites, gliosis, and loss of synaptic and dendritic density [104], are induced by an age-related accumulation of amyloid plaques in these mice. Although neuronal loss in the neocortex is not observed at 8 months of age, loss was seen in the DG of the hippocampus and other subregions in older mice [103]. These mice show spatial learning and memory impairments at 6–8 months of age in a food-rewarded four-arm spatial maze [102], MWM, and radial maze tests [105,106]. Furthermore, at 120–250 days, this model exhibits memory impairments in NOR [107] and Barns maze tests [108]. Interestingly, impairments in reversal learning in 6–9-month-old APPPS1 mice were confirmed by touchscreen visual discrimination tasks [109]. Due to the early onset of amyloidosis and the stable genetic background of this line, this mouse model is well-suited for studying the pathology of AD amyloidosis and investigating novel therapeutic strategies.

#### 5.1.6. 5XFAD

5XFAD mice overexpress the human APP and PSEN1 proteins with a total of five AD-linked mutations under the control of the mouse Thy1.2 promoter [110]. The mutations are the Swedish (K670N/M671L), Florida (I716V), and London (V717I) mutations in APP, and the M146L and L286V mutations in PSEN1. Intracellular Aβ starts to accumulate in 1.5-month-old mice, and extracellular Aβ deposition appears in 2-month-old mice [110]. 5XFAD mice accumulate more Aβ42 in the cerebrum than Aβ40, suggesting that the five FAD mutations cumulatively affect Aβ42 production. The levels of Aβ42 in this model are far higher than those found in Tg2576. However, tau hyperphosphorylation and formation of NFTs are not observed in 5XFAD mice. Astrogliosis and microgliosis present at 2 months of age, indicating that neuroinflammation occurs early in this model. Moreover, 5XFAD mice show a progressive loss of neurons, which correlates strongly with both intraneuronal Aβ42 levels and caspase-3 activation [111]. These mice exhibit progressive cognitive and memory impairments in Y-maze [112], NOR [113], FC [114], and MWM tests [112,113,114,115,116]. This mouse model could be a useful model, and it reproduces the pathology of human AD, similar to the Tg2576, APP23, and APPPS1 models.

#### 5.1.7. 3×Tg-AD

The Triple Tg (3×Tg-AD) mice overexpress the human APP protein with the Swedish mutation, the mutant PSEN1 protein, and the mutant human microtubule-associated protein tau (APP^K670N,M671L^, PSEN1^M146V^, and MAPT^P301L^) [117]. APP^K670N,M671L^ and PSEN1^M146V^ are controlled by the mouse Thy1.2 promoter. This model progressively accumulates Aβ plaques, hyperphosphorylated tau, and NFTs. Before these deposits form, synaptic dysfunction and long-term potentiation (LTP) deficits occur in this model. Intracellular Aβ deposits appear in the brain at 3 months of age in the frontal cortex and at 6 months of age in the CA1 region of the hippocampus [117] and the amygdala [118], followed by extracellular Aβ deposition progresses with aging [117]. These mice exhibit elevated levels of Aβ40 and Aβ42 with aging. Formation of intracellular NFTs, composed of hyperphosphorylated tau protein, are observed in the hippocampus at 12 months of age but not at 6 months of age. In addition, 3×Tg mice develop age-dependent synaptic dysfunction [117] and loss of tyrosine hydroxylase–positive neurons at the coeruleus locus [119]. This model does not present with the neuronal loss in the hippocampus observed in AD patients. Microglia activation is observed at 7 months of age [120]. Unlike human AD patients, no difference is observed between wild-type and 3×Tg-AD mice in white matter densities measured using magnetic resonance imaging (MRI) [121]. Behaviorally, young 3×Tg-AD mice exhibit an impairment in learning and memory deficits in MWM; these changes correlate with the accumulation of intraneuronal Aβ in the hippocampus and amygdala [118]. Additionally, this mouse model exhibits progressive cognitive and memory impairments in various behavioral tests. [118,122,123,124,125,126,127]. As the tau P301L mutation in 3×Tg-AD mice is the causative gene mutation in frontotemporal dementia but not in AD, tau pathology would differ from that observed in human AD. This model has natural tau mutations in the tau P301L background, and therefore would not express proteins that follow the amyloid cascade hypothesis. Consequently, 3×Tg-AD mice do not seem to be a mouse model that faithfully reflects AD pathology. 

### 5.2. Animal Models for Late-Onset AD

#### 5.2.1. APOE

*APOE* is a lipid metabolism–associated gene that is localized to senile plaques, vascular amyloid deposits, and NFTs in AD. The *APOE* gene is located on chromosome 19q13.2 and has three alleles, ε2, ε3, and ε4, which are present at frequencies of 8.4%, 77.9%, and 13.7%, respectively [128]. The differences between APOE2 (Cys112, Cys158), APOE3 (Cys112, Arg158), and APOE4 (Arg112, Arg158) are limited to amino acid residues 112 and 158 [129]. The presence of the *APOE ε4* allele is the strongest genetic risk factor for AD [129]. APOE4 is also associated with late-onset AD and is an important susceptibility marker for AD [129]. Additionally, APOE4 increases the neurotoxicity of Aβ, tau hyperphosphorylation, and NFTs and influences the timing and amount of amyloid deposition in the human brain [129]. The neuronal glycoprotein reelin may protect synapses against toxic Aβ through APOE receptors [130]. Therefore, APOE is a potential target for AD therapeutics. To investigate these functions, mice harboring modifications in the *APOE* gene [*APOE*-knock-in (*APOE*-KI), *APOE* knockout (*APOE* KO), and *APOE*-targeted replacement (*APOE*-TR) mice] were generated [129]. In particular, human *APOE4* KO and *APOE4*-TR mice exhibit neuronal deficits and cognitive impairments [129,131,132,133,134]. To investigate the effects of APOE on APP-induced neuropathology, *APOE*-KI/KO/TR mice were bred with APP-Tg mice [131,135,136,137]. *APOE* KO/PDAPP mice exhibited a dramatic reduction in amyloid plaque deposition at 6 months of age in the cerebral cortex and hippocampus [135]. Older human *APOE4*-TR/PDAPP mice increased the levels of insoluble APOE protein and Aβ in the brain at the same time, which decreased that of soluble APOE protein [131]. On the other hand, *APOE4*-KI/5XFAD mice exhibit delayed amyloid plaque deposition, although 5XFAD mice develop amyloid plaques by 2 months of age [137]. The delay in amyloid plaque accumulation is also observed in *APOE4*-KI/Tg2576 mice [136]. These observations suggest that APOE modulates Aβ deposition. Mice harboring *APOE* modifications crossed with APP-Tg mice could be useful tools for studying AD pathology, but these models are still under development.

#### 5.2.2. TREM2

*TREM2*, like *APOE*, is also a strong genetic risk factor for AD. *TREM2* is located on human chromosome 6p21.1 and in the IgV domain on mouse chromosome 17 [138]. In regions such as the hippocampus, spinal cord, and white matter in aged mice and elderly humans, TREM2 is highly expressed in microglia and myeloid cells [139,140]. A number of *TREM2* variants were identified as risk factors for late-onset AD [141]. Most of the variants affect the phagocytosis, maturation, and ligand affinity of TREM [142]. In the AD brain, microglia are thought to play a neuroprotective role by promoting Aβ phagocytosis, degradation, and clearance and by decreasing tau propagation. To investigate these functions, various *TREM2* KO mice were generated. *TREM2* KO mice exhibit reduced microglial activation, microgliosis, and phagocyte levels [143], suggesting that TREM2 modulates the inflammatory response and phagocytosis in microglia. Expression of TREM2 increases in AD patients [144,145]. Adeno-associated virus-mediated soluble TREM2 (sTREM2) expression reduces amyloid plaque load and rescues spatial memory and LTP deficits in 5XFAD mice [116]. sTREM2 enhances microglial proliferation, migration, clustering in the vicinity of amyloid plaques, and the uptake and degradation of Aβ, suggesting that sTREM2 may protect against AD pathology [116]. To further understand the role of microglia in AD, *TREM2* KO mice were crossed with APP-Tg mice [146,147,148,149]. *TREM2* KO/APPPS1 mice exhibit a reduction in inflammation and the accumulation of amyloid and tau [146], suggesting that TREM2 is involved in AD pathology. Hence, mice harboring *TREM2* modifications crossed with APP-Tg mice could be used to investigate novel AD therapeutic approaches that target the microglia.

**Table 2:** Summary of representative APP-Tg and *APP*-KI mice in AD model mice.

Mouse Line	Promoter	Transgene Mutation	Amyloid Plague-Deposits	Hyperphosphorylated Tau	NFTs	Reference
Tg2576	Hamster Prion Protein	APP Swedish mutation	11–13 months	Not detected	Not detected	[70]
APP23	Mouse Thy1	APP Swedish mutation	6 months	6 months	Not detected	[78]
PDAPP	Platelet-derived growth factor-β	APP Indiana mutation	6–9 months	14 months	Not detected	[88,89]
TgCRND8	Hamster Prion Protein	APP Swedish + Indiana mutations	3–5 months	7–12 months	Not detected	[93,94]
APPPS1	Mouse Thy1 (APP, PS1)	APP Swedish + PS1 L166P mutations	2–3 months	8 months	Not detected	[102]
5XFAD	Mouse Thy1.2 (APP, PS1)	APP Swedish + Florida + London + PS1 M146V + L286V mutations	1.5 months	Not detected	Not detected	[110]
3×Tg-AD	Mouse Thy1.2 (APP, Tau) and endogenous (PS1)	APP Swedish + PS1 M146V + Tau P301L mutations	3–6 months	12 months	12 months	[117]
*APP*-KI	Endogenous APP	APP Swedish + Iberian + Arctic mutations	2 months	Not detected	Not detected	[150]

## 6. The Next Generation of Mouse Models and New Approaches for AD Treatment

Although APP-Tg mice have been used over the past 5 years to develop new AD therapeutic strategies using behavioral tests related to cognition and memory formation (Table 3), these mouse models have intrinsic problems that induce artificial phenotypes. In these models, some APP fragments, including Aβ, are over-produced. For example, APP23 mice have high levels of APP intracellular domain (AICD) [151] and C-terminal fragment β (CTF-β) [152]. These APP fragments may be involved in various functions including the control of Aβ degradation, cell death, and γ-secretase activity. Unlike AD patients, Tg2576 and APP23 mice exhibit the accumulation of different Aβ species, including Aβ40 and Aβ42. Aβ42 is the predominant species accumulated in the brain of AD patients. There are also differences in the generation of hyperphosphorylated tau among APP-Tg mice, although APP-Tg mice, with the exception of 3×Tg-AD mice, just overexpress the mutant human APP protein. These differences could be caused by overexpression of APP transgenes with artificial mutations [150].

To overcome these undesired problems, an *APP* gene knock-in (*APP*-KI) mouse with the Swedish (KM670/671NL), Beyreuther/Iberian (I716F), and Arctic mutations was generated [150]. This model overproduces Aβ42 without overexpressing APP. *APP*-KI mice exhibit excessive Aβ deposition in the cortex and hippocampus with age. Additionally, microgliosis and astrocytosis are observed at 9 months of age. Moreover, synaptic alternation and memory impairment, similar to what is seen in AD patients, are observed in these mice. On the other hand, as with other AD models, this model does not have tau pathology, NFTs, neurodegeneration, or massive neuron loss [153]. Therefore, this mouse model should be used to study preclinical AD. Various classical behavioral tasks to evaluate cognitive and memory functions at the early stage were performed with this model [154,155,156,157]. Behavioral impairments were not observed in young *APP*-KI mice, suggesting that these tests are unsuitable for evaluating the preclinical and early-onset stages of AD. 

In recent years, it has become clear that early detection of AD is important for effective interventions [158]. Clinical studies demonstrate that early treatment initiation with the currently existing medications provides a more effective clinical benefit than later initiation of such therapy [159,160]. To better evaluate cognitive function in psychiatric and neurodegenerative disorders, new technology using touchscreen-based tasks was developed [161,162,163]. This technology assesses attention, learning, and memory in animals and humans. We showed that touchscreen-based tasks can detect cognitive impairment in *APP*-KI mice at 4–5 months of age [164]. This was the first report to show the benefit of these methods in detecting the early stage of cognitive impairment in an AD-linked *APP*-KI mouse model. Therefore, this advanced technology would detect preclinical AD-like behaviors in AD mouse models and would aid in the search for new therapeutic approaches to prevent AD progression. 

Although various studies have focused on the relationship between AD and microglia, Sobue et al. [165] recently performed RNA sequencing using magnetic-activated cell sorting to analyze the microglia of three neurodegenerative disease-associated model mice: *APP*-KI with amyloid pathology, rTg4510 with tauopathy, and SOD1^G93A^ with motor neuron disease. RNA sequencing was also used to analyze preclinical AD human precuneus, and the results were compared to those of a control group. Interestingly, the loss of unique microglial homeostatic genes in the progression of AD correlates with the severity of neurodegeneration. Moreover, RNA sequencing results for the human precuneus samples led us to conclude that amyloid pathology in early-onset AD induces loss of microglia and oligodendrocyte function. Further advances in these beneficial tools are expected in the future.

**Table 3:** Summary of reference with learning- and memory-associated behavioral tests in popular AD model mice over the past 5 years.

Behavioral Tests	Test Significance	Significant Difference								
		Tg2576	APP23		PDAPP	TgCRND8	APPPS1	5XFAD	3xTg-AD	*APP*-KI
Y-maze	Short-term working memory	Yes: [166] No: [125]	―		―	―	―	Yes: [112]	Yes: [125,126]	No: [156]
Hole board	Reference and working memory	Yes: [167] No: [167]	―		―	―	―	―	―	―
Open-field foraging task	Working memory	―	―		Yes: [168]	―	―	―	―	―
Object in place task	Spatial recognition memory	No: [169]	―		―	―	―	―	―	―
Object place recognition	Short-term memory	Yes: [125]	―		―	―	―	―	Yes: [125]	―
Object-place association task	Recognition memory	―	―		―	Yes: [98]	―	―	―	―
Spatial object location task	Recognition memory	―	―		―	Yes: [98]	―	―	―	
	Spatial memory									No: [170]
Novel object recognition	Recognition memory	Yes: [171] No: [169]	Yes: [86]		―	Yes: [98,100]	Yes: [108]	Yes: [113]	Yes: [122,123]	No: [156]
	Hippocampal-dependent episodic memory	Yes: [125]							Yes: [125]	
Social preference social novelty	Social memory	―	―		―	―	―	―	―	No: [157]
Fear conditioning	Associative memory	No: [172]	No: [173]		―		―	―	―	
	Contextual fear memory					Yes: [99]				Yes: [170]
	Tone-cued fear memory									No: [170]
	Non-hippocampal-dependent auditory fear memory					Yes: [99]				
	Fear learning									No: [155]
Fear conditioning context discrimination	Context discrimination learning and memory	Yes: [73]	―		―	―	―	―	―	―
Passive avoidance	Contextual learning and memory	―	―		―	―	―	―	Yes: [122]	―
	Learning and memory								Yes: [126]	
Eight-arm radial maze	Working memory	―	Yes: [84]	―			―	―	―	―
	Spatial learning and memory					Yes: [100]				
Banes maze	Spatial learning and memory	―	―	―			Yes: [108]	―	―	Yes: [155]
	Spatial navigation memory					Yes: [98]				
Morris water maze	Spatial learning and memory	Yes: [124,166,171,174] No: [175]	Yes: [85] No: [86]	―		Yes: [99]	―	Yes: [112,113,115]	Yes: [122,124,125,126,127]	
	Spatial recognition memory	No: [174]								
	Spatial reference memory									No: [156,157,164]
Spatial reversal learning	Flexibility and impulse control	―	―	―		―	―	―	―	No: [155]
Location discrimination	Pattern separation	―	―	―		―	―	―	―	Yes: [164]
Different object –location paired-associate learning	Paired-associative memory	―	―	―		―	―	―	―	Yes: [164]
Visual discrimination, reversal learning	Cognitive flexibility	―	―	―		―	Yes: [109]	―	―	No: [164]

## 7. Pharmacological Interventions in AD Animal Models

Over the years, various amyloid cascade hypothesis–related AD model mice were generated to develop new therapeutic drugs for AD treatment. Although the acetylcholinesterase inhibitors donepezil, galantamine, and rivastigmine, and the *N*-methyl-*D*-aspartate receptor inhibitor memantine are FDA-approved therapeutics, these drugs are only able to delay the progression of AD. All of the approved drugs ameliorate cognitive deficits and decrease Aβ deposits in Tg2576 mice [176,177,178,179] and APP23 mice [180], which is consistent with clinical trial results [181,182,183,184]. Therefore, APP-Tg mice should be useful tools for developing new therapeutics aimed at suppressing the progression of AD. Drug repositioning is an attractive method for finding novel therapeutics. The nonsteroidal anti-inflammatory drug ibuprofen reduces amyloid deposits and inflammation [185] and increases memory and synaptic plasticity in Tg2576 mice [186] and 3×Tg-AD mice [187], but these effects are not seen in 5XFAD mice [188]. However, ibuprofen has both positive [189] and negative effects [190] in clinical trials. The peroxisome proliferator-activated receptor γ agonists rosiglitazone and pioglitazone also ameliorate hippocampus-dependent memory impairment in Tg2576 mice [73,191], APP/PS1 mice [192], and 3×Tg-AD mice [193], but the results from clinical trials were not consistent with those from the animal experiments [194,195,196,197]. Multiple AD clinical trials using statin drugs, which decrease blood cholesterol, were performed. However, a phase IV clinical trial with the statin simvastatin was unsuccessful [198]. As a result, further studies with statins are needed to understand their mechanism of action in AD treatment. Rifampicin also reduces Aβ accumulation in Tg2576 mice [199], but little is known about the mechanism by which it modulates AD-related amyloid deposits. 

Several promising novel therapeutic candidates for the treatment of AD have emerged. BACE and γ-secretase inhibitors suppress the production of insoluble Aβ, reduce Aβ deposition, and ameliorate cognitive impairments in Tg2576 [200], PDAPP [201], and APPswe/PSEN1dE9 mice [202]. However, because these inhibitors aggravate cognitive impairments and have side effects, no inhibitors passed preclinical trials [203,204,205]. The usefulness of Aβ42 vaccination was first observed in PDAPP mice [206]. PDAPP mice that received this vaccine showed reduced Aβ depositions. The vaccination ameliorated memory abnormalities in APP+PS1 [207] and TgCRND8 mice [96]. The vaccination was also used to develop anti-Aβ antibodies, and their administration induced a dramatic reduction in Aβ and recovery of memory deficits in Tg2576 [208,209] and PDAPP [210,211] mice without side effects. The anti-Aβ antibodies were administrated in clinical trials, but patients developed meningoencephalitis, and the trial was discontinued [212]. However, a follow-up study with AD patients from this trial who experienced negative side effects shows that patients have long-term clinical benefits such as amelioration in cognitive impairment and a reduction in CSF tau levels [213]. This led to the development of humanized anti-Aβ antibody drugs [214,215,216,217] and Aβ vaccines [218], and several clinical trials with anti-human Aβ monoclonal antibodies were performed [219,220]. Unfortunately, most of the trials were either unsuccessful or did not show a dramatic improvement in AD pathology [219,220]. Given that the amyloid cascade hypothesis states that Aβ is first deposited extracellularly 20–30 years before the onset of cognitive and memory deficits in the FAD brain, immunotherapy trials may have been started too late in the clinical phase, when too much Aβ had accumulated, and the Aβ cascade was already irrevocably initiated [219]. Despite these controversies, development of these antibody drugs is ongoing under a conventional clinical trial. Therefore, it is expected that new trials will be initiated earlier in the course of AD and that these antibodies will play a central role in future treatment strategies [219]. Hyperphosphorylated tau vaccines were also developed using AD model mice that overexpress mutant human tau, but their effects have yet to be confirmed in clinical trials [221,222]. Given the challenges and a large number of failures, development of new therapeutic drugs for AD treatment is strongly desired.

## 8. Conclusions

We introduced the molecular mechanisms of AD, including the amyloid cascade and tau propagation hypotheses, and biomarkers of AD. We also described representative APP-Tg mice in brief. Moreover, we indicated whether APP-Tg mice are appropriate as an AD animal model, concluding that they should be a partial model of AD because the phenotypes of these mice differ from those of AD patients. Although AD has been studied for many years, no effective therapeutic strategies for AD have been found. In terms of translational research, the gap between basic and clinical drug discovery screening methods may be one of the reasons why candidate drugs do not easily become therapeutic drugs for AD. In order to elucidate the mechanisms of AD onset, therapeutic strategies that initiate from a new point of view or unconventional methods are desired. Namely, new animal models that faithfully recapitulate AD pathology and new technologies that accurately detect AD symptoms are required. To solve some of these problems, new diagnostics and therapeutic approaches, such as *APP*-KI mice, touchscreen-associated testing techniques, and anti-Aβ drugs, were developed. As the development of these novel approaches increases, it is hoped that AD treatment targets that reduce or eliminate Aβ and NFT deposits will emerge. In addition, the accumulating studies that focus on the more common AD treatment approaches will be useful for supporting the detection of biochemical parameters and accurately monitoring AD progression.

## Figures and Tables

**Table 1:** Summary of representative biomarkers in AD.

Modality	Type	Reference
CSF	Aβ	[36]
	Tau	[38,39,40]
	Aβ oligomers	[42,43]
PET	Amyloid	[44,45,46,47]
	Tau	[48,49]
	Microglia	[50,51]
Blood	Aβ	[55,56,57,58]
	Secondary structure of Aβ	[59]
	Tau	[60,61,62]
	IL-8	[63]
	Neurofilament light	[64]
	MicroRNAs	[65]
	IGF-1 and IGF-binding protein-3	[66]
	APOA-I, C3, and transthyretin	[67]

## Data Availability

Not applicable.
